# A Novel Glucose Biosensor Based on Hierarchically Porous Block Copolymer Film

**DOI:** 10.3390/polym10070723

**Published:** 2018-07-02

**Authors:** Teng Guo, Jiefeng Gao, Xiang Qin, Xu Zhang, Huaiguo Xue

**Affiliations:** School of Chemistry and Chemical Engineering, Yangzhou University, Yangzhou 225002, China; a6800000@126.com (T.G.); 18705274178@163.com (X.Q.); -XuZhang@163.com (X.Z.)

**Keywords:** hierarchical porosity, block copolymer film, glucose detection

## Abstract

Enzymatic biosensors are widely used in clinical diagnostics, and electrode materials are essential for both the efficient immobilization of enzyme and the fast electron transfer between the active sites of enzyme and electrode surface. Electrode materials with a hierarchically porous structure can not only increase the specific surface area but also promote the electron transfer, facilitating the catalysis reaction. Block copolymer is a good candidate for preparation of film with a hierarchically porous structure due to its unique characteristics of self-assembly and phase separation. In the current work, hierarchically porous block copolymer film containing both micropores and nanopores was prepared by spinodal decomposition induced phase separation. The resultant copolymer film was adopted as the electrode material to immobilize glucose oxidase (GOx) for construction of an enzyme biosensor. Scanning electron microscopy (SEM), contact angle (CA) measurements, and Fourier-transform infrared (FTIR) and electrochemical impendence spectroscopy (EIS) were adopted to investigate the microstructure of the as-developed biosensor. Results demonstrated that the hierarchically porous block copolymer film offered a favorable and biocompatible microenvironment for proteins. These as-prepared glucose biosensors possessed a wide linear range (10–4500 μM), a low detection limit (0.05 μM), quick response (2 s), excellent stability, and selectivity. This work demonstrates that hierarchically porous block copolymer film is a good matrix candidate for the immobilization of the enzyme and provides a potential electrode material to construct novel biosensors with excellent performance.

## 1. Introduction

A biosensor, which consists of confident biological component coupled with a transducer device, can convert a biochemical signal into an amplified electrical signal [1,2,3]. Currently, biosensors play an important role in the areas of clinical diagnostics [4,5]. Up to now, enzymatic biosensors based on the immobilized enzymes are the more frequently used biosensor [6,7,8]. Thus, the immobilization of enzymes is a key step in the preparation of biosensors. The immobilization of enzymes can maintain the structural integrity of enzymes for a longer time [9]. Therefore, it is of high importance to achieve the efficient immobilization of enzymes for the construction of high performance biosensors. 

In general, it is crucial to efficiently immobilize enzymes while at the same time maintaining effective electron transfer between the active sites of enzymes and the electrode surface. Various conducting materials have been used as electrode materials, like graphene [10], carbon nanotubes [11], metal oxides [12], conducting polymer [13], and so on. For example, copper oxide nano-wire arrays were in situ grown on the three-dimensional copper foam, which was then used to construct the glucose biosensor [14]. The structures of nano-wire arrays and nano-flowers increased the specific surface area of the material, and thus enhanced the electrocatalytic performance of the integrated electrode. The as-developed biosensor exhibited excellent performance with a sensitivity of 32,330 mA·mM^−1^·cm^−2^, a low detection limit of 20 nM (S/N = 3), excellent selectivity, reproducibility, and stability. On the other hand, porous structure, especially the hierarchically porous structure containing micropores and nanopores, is introduced into the electrode material in order to increase the reaction sites and enhance the electron transfer [15].

Block copolymers provide many attractive possibilities in designing hierarchically porous materials [16,17,18]. Spontaneous structural organization over roughly mesoscopic length scales could be obtained in consideration of the amazing phase-separated morphologies and easy accessibility through the control of block chemistry, molecular architecture, and block length. In addition, nano-porous materials with uniform pore shapes and sizes were obtained by selectively etching the domains forming by a specific block(s) [19]. For instance, Schöttner et al. [20] combined the self-assembly and non-solvent-induced phase separation to prepared the asymmetric polystyrene-*block*-poly(2-hydroxyethyl methacrylate) (PS-*b*-PHEMA) film possessing a perfect hexagonal lattice structure, with apore diameter of 24.7 ± 2.5 nm. Moreover, vertically oriented pores were present on the top surface of the asymmetric film.

In the present work, we prepared hierarchically porous block copolymer film containing micropores and nanopores by macro- and meso-phase separation, which was induced by spinodal decomposition. Subsequently, the resultant film was adopted as the electrode material to immobilize glucose oxidase (GOx). The as-developed glucose biosensor displayed a long linear range (10–4500 μM), a low detection limit (0.05 μM), and quick response (2 s). Moreover, the selectivity and stability were also investigated. This work illustrates that the hierarchically porous block copolymer films as electrode materials have great potential applications in the field of high performance enzyme biosensors.

## 2. Materials and Methods

Polystyrene-*block*-poly(4-vinyl pyridine) (PS_9w_-*b*-P4VP_5w_) was obtained from Polymer Source Inc. (Quebec, QC, Canada); polyethylene glycol (PEG, average *M_n_*~400 g·mol^−1^) was provided from Aladdin Chemical Reagent Co.(Shanghai, China); anisole was received by Lingfeng Chemical Reagent Co. (Shanghai, China); glucose oxidase (GOx) from *Aspergillus niger* (powder, light brown, ≥100,000 U/g) was supplied by Sigma-Aldrich Chemical Co., Ltd. (St. Louis, MO, USA); d-glucose, KH_2_PO_4_, and K_2_HPO_4_ were purchased from Sinopharm Chemical Reagent Co. (Shanghai, China). d-glucose solution was prepared one day in advance and mutarotated at room temperature. All liquid reagents used for the preparation of block copolymer film were carefully dried over molecular sieves (3A). All of the aqueous solutions in the process of electrochemical measurements were prepared with twice-distilled water. All the other chemical reagents were analytical grade and used without further purification. The platinum (Pt) electrodes were successively polished to a mirror-like surface with 0.5 μm and 50 nm alumina slurry and then rinsed thoroughly by twice-distilled water. To get rid of the loosely adsorbed alumina slurry, the electrodes were washed with anhydrous ethanol for 3 min in an ultrasonic bath, rinsing thoroughly by twice-distilled water followed by drying with a stream of nitrogen. Phosphate buffer (PB) solution was transferred to 6.5 with 0.1 M of KH_2_PO_4_ and K_2_HPO_4_ in total. 

The morphologies of PS-*b*-P4VP film and GOx/PS-*b*-P4VP composite film were observed with scanning electron microscopy (SEM, Zeiss_Supra55, Carl Zeiss AG, Oberkochen, Germany). Prior to SEM observation, the films were sputter coated for 35 s with gold to avert charge accumulations during the test. The measurements for the contact angles (CA) were carried out with a video optical contact angle measuring instrument (OCA20). Attenuated total reflectance Fourier transform infrared (ATR-FTIR, Agilent Technologies Inc., Santa Clara, CA, USA) spectrum was recorded in the range of 500−3600 cm^−1^ on a Cary610 ATR-FTIR spectrometer. Electrochemical impedance spectroscopy (EIS, Pgstat 302N, Metrohm, Switzerland) was adopted to determine the charge transfer resistance of bare Pt electrode, PS-*b*-P4VP/Pt, and GOx/PS-*b*-P4VP/Pt. EIS was studied in 0.1 M PB containing 5 mM Fe(CN)_6_^3+^/^4+^ and 0.1 M KCl with a frequency range of 0.05 Hz to 100 kHz. Electrochemical workstation (CHI660A) was used for amperometric measurements. A three-electrode system with a working electrode (Pt, φ=3 mm), a counter electrode (platinum sheet, Pt sheet, 10×10 mm^2^), and a reference electrode (saturated calomel electrode, SCE) was employed for the electrochemical measurements.

### 2.1. Preparation of Hierarchically Porous PS-b-P4VP Film

**Figure 1:**
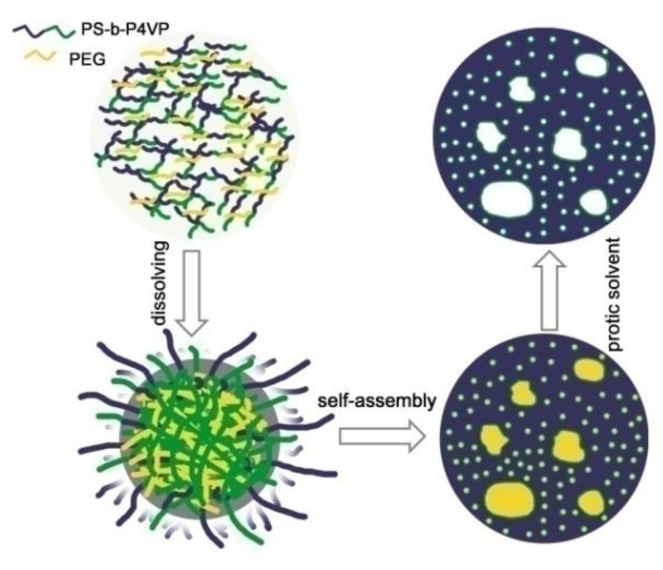
Schematic illustration for the fabrication of the hierarchically porous PS-*b*-P4VP film.

0.32 g of PS-*b*-P4VP and 0.28 g of PEG were dissolved in 5.4 g of anisole at 40 °C for 12 h to form a homogeneous solution, followed by filtration with a 0.22 µm PTFE syringe filter to remove impurities. Then, the polymer solution was dropped onto the surface of electrode and subsequently heated at 130 °C for 2.5 h. The polymer solution was solidified with evaporation of the solvent, and a white copolymer film was generated on the electrode surface. Then the copolymer film deposited on the electrodes was immersed in ethanol and gently stirred for 2 h to remove the PEG from the BCP/PEG co-assembled structures and the macro-phase separated PEG-rich domain. Finally, the hierarchically porous PS-*b*-P4VP film was obtained. The schematic demonstration for the fabrication of hierarchically porous block copolymer film is shown in Figure 1.

### 2.2. Construction of Glucose Biosensor

1.5 mg GOx was dissolved with 1.5 mL PB solution (pH 6.5) to form a uniform solution. Afterwards, 8 μLof the solution was dropped on the surface of the pretreated Pt electrode; and dried in a refrigerator. Then the electrodes were exposed to 25% glutaraldehyde vapor for 15–20 min so as to avoid the leakage of the immobilized enzyme molecules. Moreover, the GOx/PS-*b*-P4VP/Pt was rinsed thoroughly with twice-distilled water to remove the loosely adsorbed GOx molecules. All the as-prepared electrodes were stored at 4 °C in a refrigerator before use. All the experiments were carried out at room temperature, unless otherwise specified.

## 3. Results and Discussions

### 3.1. Characterizations of the Hierarchically Porous Film and Glucose Biosensor

**Figure 2:**
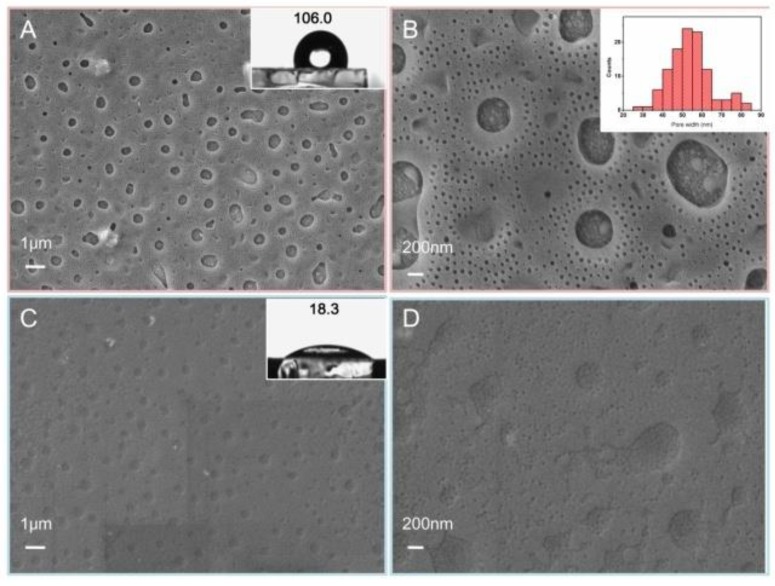
SEM images of PS-*b*-P4VP film (**A**,**B**) and GOx/PS-*b*-P4VP composite film (**C**,**D**) with different magnification; contact angle images of PS-*b*-P4VP film (inset **A**) and GOx/PS-*b*-P4VP composite film (inset **C**); size distribution of nanopores in PS-*b*-P4VP film (inset **B**).

The surface morphologies of PS-*b*-P4VP film before and after immobilization of GOx are shown from the SEM images in Figure 2. PS-*b*-P4VP film (Figure 2A,B) displays a hierarchically porous structure including micropores and nanopores. Moreover, the pore diameter of nanopores is larger than 25 nm (inset in Figure 2B), which is beneficial for the immobilization of enzyme with the size of 10–100 nm. Furthermore, the hierarchically porous structure not only increases the specific surface area and active sites of catalysis reaction but also enhances the mass transfer during the reaction. After anchoring GOx into the copolymer matrix, the pores in the film disappear strikingly, indicating the enzyme is successfully immobilized on the porous PS-*b*-P4VP film (Figure 2C,D). In addition, the hydrophilicity is a vital factor affecting the biocompatibility of a host matrix for enzyme, which was evaluated by the static contact angle (CA). To some extent, electrode materials with more hydrophilicity can provide a more favorable microenvironment for the immobilized enzymes. As is shown in the inset of Figure 2A,C, the CAs of PS-*b*-P4VP/Pt and GOx/PS-*b*-P4VP/Pt are measured to be 106.0° and 18.3°, respectively. After introduction of GOx, the CA of the electrode material decreases remarkably on account of the abundant hydrophilic groups of enzyme molecules such as amino and carboxyl groups, indicating successful immobilization of GOx into the hierarchically porous block copolymer film.

**Figure 3:**
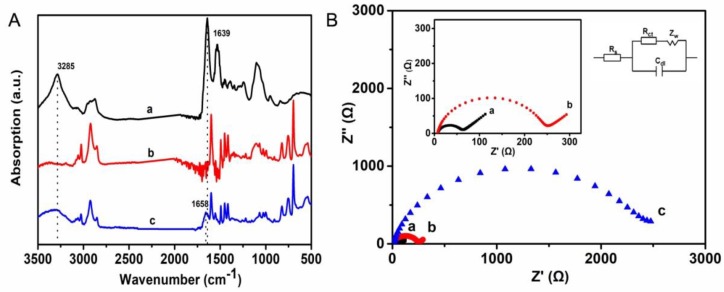
(**A**) The ATR-FTIR spectrum of GOx (**a**), PS-*b*-P4VPfilm (**b**), and GOx/PS-*b*-P4VP composite film (**c**); (**B**) Nyquist plots of EIS for bare Pt (**a**), PS-*b*-P4VP/Pt (**b**), and GOx/PS-*b*-P4VP/Pt (**c**) in 0.1 M PB containing 0.1 M KCl and 5 mM [Fe(CN)_6_]^3+^/^4+^ for a frequency range of 0.05 Hz to 100 kHz. Inset **B**: Equivalent circuit applied to fit the impedance spectra.

The ATR-FTIR spectroscopy was utilized to characterize the interaction of the immobilized GOx and the matrix, which is shown in Figure 3A. For native GOx (curve a), a relatively broad peak centered at 3285 cm^−1^ is related to the N–Hand O–H stretching vibrations in protein. Moreover, the peaks at 1639 and 1530 cm^−1^ can be attributed to the characteristic absorption peaks of amide I (1700−1600 cm^−1^) and amide II (1600−1500cm^−1^) bands of protein [21]. The absorption peaks at 2921 and 2849 cm^−1^ in curve b are associated with the characteristic absorption peaks of alkyl I (2925 cm^−1^) and alkyl II (2850 cm^−1^) for the block copolymer film. The absorption peaks at 1599, 1494, and1451 cm^−1^ are ascribed to the stretching vibration peaks of benzene ring. The absorption peaks at 752 and 694 cm^−1^ are assigned to pyridine and benzene, respectively. Compared with curve a and b, the GOx/PS-*b*-P4VP composite film possesses the characteristic absorption peaks of both GOx and PS-*b*-P4VP (curve c) as mentioned above, indicating that GOx has been successfully immobilized on the PS-*b*-P4VP film. In addition, the peaks for amide I and II bands shifts from 1639 to 1658 cm^−1^, indicating the intermolecular interaction between enzyme and the hierarchically porous PS-*b*-P4VP film.

Electrochemical impedance spectroscopy (EIS) is a powerful electrochemical method that can provide electrochemical information of the electrodes. It can reflect the electron transfer resistance and the diffusion of redox-active compounds within solution and electrode interface [6]. Figure 3B shows the Nyquist plots obtained for each electrode surface. In the equivalent circuit, the electrode series resistance *Rs* comprises the resistance of the electrode and electrolyte resistance, *C_dI_* is the constant phase element related to the double layer capacitance, and the *R_ct_* represents the charge transfer resistance. All electrodes after modification display a semicircle resulting from *R_ct_* in the high frequency region, followed by a linear region known as Warburg impedance *Z_w_* in the low-frequency region, and the *Z_w_* corresponds to the diffusion process. The value of *R_ct_* can be obtained via the diameter of semicircle. The *R_ct_* values of bare Pt, PS-*b*-P4VP/Pt, andGOx/PS-*b*-P4VP/Pt are 73, 252, and 2453 Ω, respectively. Compared with the bare Pt, PS-*b*-P4VP/Pt has a relatively slight increase in the *R_ct_*, indicating that the PS-*b*-P4VP film retains the great transference of electron, which may be ascribed to the hierarchically porous structure. In addition, an obvious increase in the resistance and the weak diffusion process of the GOx/PS-*b*-P4VP/Pt can be observed when GOx is immobilized in the copolymer film, which results from the hindrance of the macromolecular structure of GOx to the electron transfer. This result is in agreement with that of Shan [22] and Su [23]. Thence, the GOx was successfully immobilized onto the PS-*b*-P4VP film.

### 3.2. Optimization of the Glucose Biosensor

**Figure 4:**
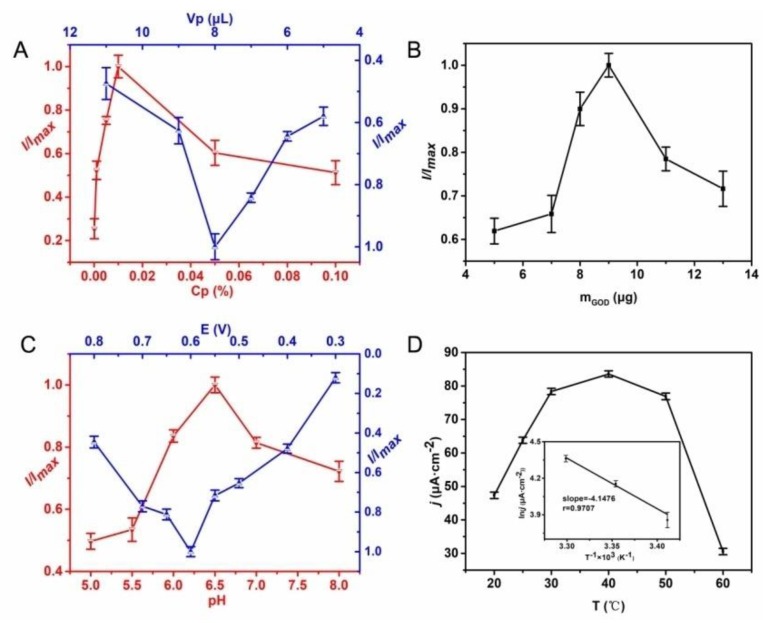
(**A**) Effect of the polymer concentration (red line) and the volume of casting solution (blue line). (**B**) Effect of the amount of GOx. (**C**) Effect of pH (red line) and applied potential (blue line). (**D**) Effect of temperature on the current response of GOx/PS-*b*-P4VP/Pt in 10 mL of 0.1 M PB solution of pH 6.5 for 10 μM glucose.

The performance of enzyme biosensor relates to many factors, for example, the thickness of bio-composite film and external factors. In this work, the thickness of bio-composite film mainly relies on the polymer solution and the amount of enzyme. In general, a higher concentration of polymer results in a thicker film that may hinder the fastelectron transfer. Figure 4A shows the effect of current response with different polymer concentration and volume. When the concentration of polymer is lower than 0.01%, the current response increases gradually with the increase of the concentration and reaches the maximum response at 0.01 wt %. Therefore, 0.01 wt % is considered as the optimal polymer concentration to achieve a large current response. The current response is also affected by the solution volume, as shown in blue line of Figure 4A. With increasing the volume of polymer solution from 5 to 8 μL, the current response of the biosensor increases from 3.47 to 5.98 μA. Afterwards, the current response begins to decrease. Generally speaking, a low volume of casting solution cannot cover the surface of electrode while a large volume of casting solution can lead to the overflow of the solution out the electrode surface; therefore 8 μL was the favorable volume. In addition, the amount of enzyme also posed a great effect on the performance of enzyme biosensor. As shown in Figure 4B, the current response increases with the amount of enzyme; and when the amount of enzyme is larger than 9 μg, the current response starts to fall. This phenomenon may be explained as the active sites of enzyme being easily shielded impairing the catalytic reaction with the increase of the enzyme amount. As a result, 9 μg of GOx was adopted to construct the glucose biosensor in the following experiments.

External factors—such as pH, temperature and applied potential—can also influence the electrochemical performance of enzyme biosensors. Among them, pH and temperature determine the enzymatic electrocatalytic activity and stability, while the applied potential affects the sensitivity and selectivity of the biosensor. The influence of pH on current response is displayed in Figure 4C. The current response has a sharp rise with the pH changed from 5.0 to 6.5, and then declines with further increase of the pH. Thus, pH 6.5 is considered as the optimum condition for the immobilized GOx in this work. As known, the activity and stability of enzyme severely depend on the temperature too. As shown in Figure 4D, the current response ascends to 83.61 μA·cm^−2^ with the increase of temperature from 20 to 40 °C. Then the current response descends when the temperature rises to 60 °C. We carried out the following experiments at 25 °C, because the protein unfolding occurred and long term stability of the biosensor was sacrificed at a relatively high temperature. However, the measurement of relationship between current response and temperature is not meaningless. The apparent activation energy (*Ea*) of the reaction can be calculated according to the Arrhenius relationship

(1) $$\ln k=-\frac{Ea}{R\times T}+\ln A$$

In the formula, *R* is the molar gas constant, *T* is the thermodynamic temperature and *A* is pre-exponential factor. ln*k* could be replaced with ln*I*. Then *E_a_* was obtained by the slope of the line (see the inset of Figure 4D), which was calculated to be 34.48 kJ·mol^−1^. Moreover, the influence of applied potential on the sensitivity and selectivity of the enzyme biosensor is displayed in Figure 4C. The current response increases rapidly as the potential increases from 0.3 to 0.6 V, which may be caused by the increased driving force for the reaction. However, further increase of the positive potential results in the fall of current response. Considering that some interfering compounds are easily oxidized at high potentials, thereby lessening the selectivity of biosensors [6], 0.6V was determined as the optimal applied potential in the following experiments.

### 3.3. Performance of the Proposed Glucose Biosensor

**Figure 5:**
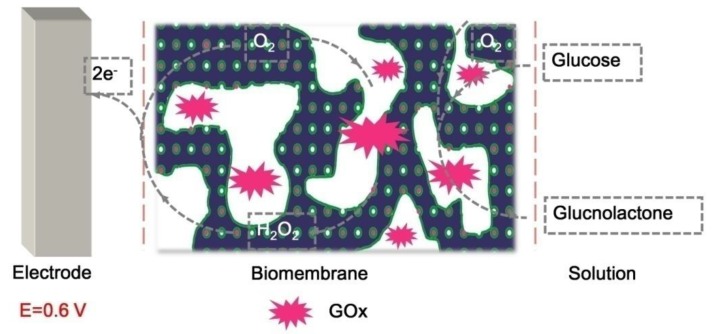
The schematic reaction mechanism of glucose biosensor.

**Figure 6:**
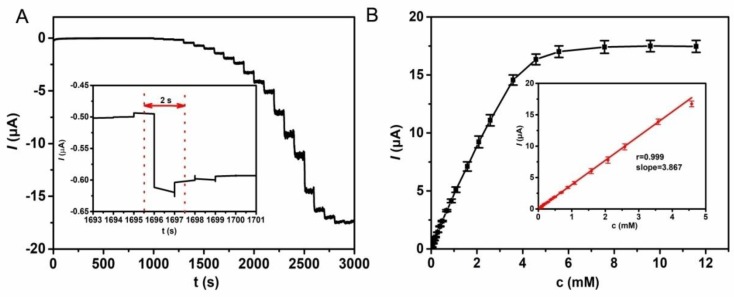
(**A**) Curve of steady-state response after injecting glucose into 10 mL of 0.1 M PB solution under fast stirring; Potential: 0.6 V. Inset **A** Partial magnification of Figure 6A. (**B**) Steady-state current-time response of the GOx/PS-*b*-P4VP/Pt by increasing glucose concentrations. Inset **B**: Calibration curve of steady-state current–time response.

As is known, GOx can catalyze the oxidation of glucose to produce gluconolactone and hydrogen peroxide [24,25]. The electrocatalytic process is demonstrated in Figure 5. In the catalysis reaction, oxygen acts as the role of an electron acceptor, and the electron can be transported to the electrode quickly resulting in the current response, which is ascribed to the hierarchically porous structure of the block copolymer film. Figure 6A gives the typical amperometric response at the GOx/PS-*b*-P4VP/Pt by successive injection of glucose to the air-saturated PB solution. The first current step for the response appears when adding 10 μM glucose solution into the PB solution. The steady-state current is achieved within 2 s, suggesting a high electrocatalytic oxidation and fast electron conduction behavior (inset in Figure 6A). The quick current response may result from the hierarchically porous structure of block copolymer film. The current response rises linearly with the increase of the glucose concentration in the range of 10 to 4500 μM. It can also be found that the detection limit is 0.05 μM at a signal-to-noise of 3. From the Figure 6B, the sensitivity of GOx/PS-*b*-P4VP/Pt can be calculated to be 54.74 mA·M^−1^·cm^−2^. The biosensing performance based on the GOx/PS-*b*-P4VP/Pt is compared with the results reported in the literature, which is shown in Table 1. It can be found the GOx/PS-*b*-P4VP/Pt exhibits obvious predominance in the linear range, detection limit, and response time compared with other modified electrodes. Furthermore, the apparent Michaelis–Menten constant (*Km*) was calculated to be 3 mM for the glucose biosensor via Lineweaver–Burk plot. For the reason that *Km* demonstrates the activity of enzyme and affinity between enzyme and immobilization matrix, such low value of *Km* indicates that the hierarchically porous PS-*b*-P4VP film provides a biocompatible micro-environment for enzyme; and these materials are considered as a potential electrode material to immobilize enzyme.

### 3.4. Interference Study and Stability of the Biosensor

**Figure 7:**
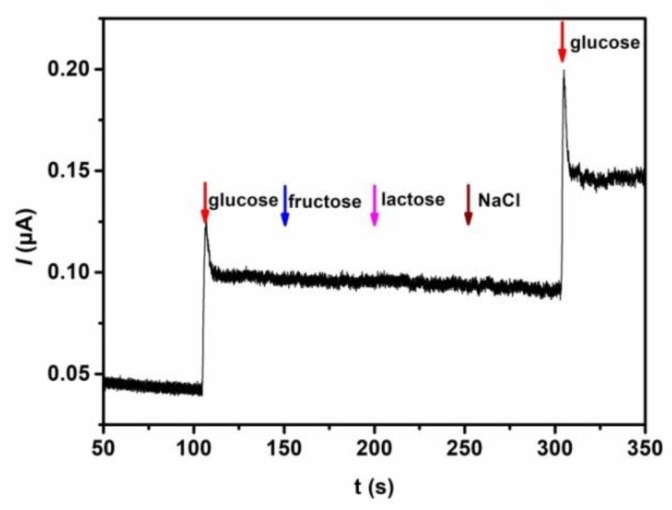
Amperometric response of the GOx/PS-*b*-P4VP/Pt to10 μM glucose, fructose, lactose, NaCl, and glucose.

**Figure 8:**
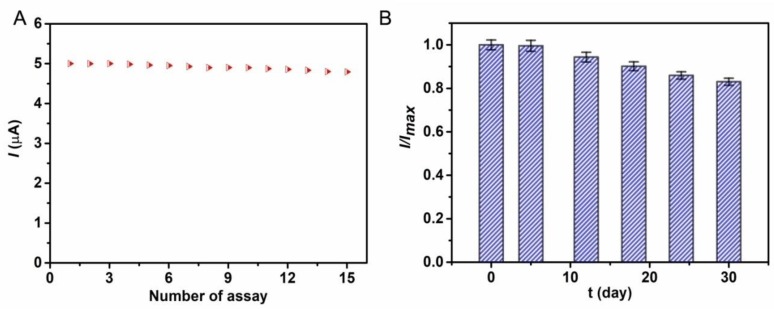
(**A**) The operational stability and (**B**) long-term stability of GOx/PS-*b*-P4VP/Pt response to 10 μM glucose under the optimal conditions.

The selectivity of the biosensor for quantitative detection of glucose was examined by using glucose, fructose, lactose, and NaCl as analytes. As shown in Figure 7, an obvious current response appears when 10 μM glucose solution is added to the electrolyte solution. However, with successive injection of fructose, lactose, and NaCl to the system, the current response is unchanged. When the glucose is added to the solution again, the current response appears once again and the increased current value is close to the initial current value, which indicates the excellent anti-interference ability of the biosensor. In addition, the reproducibility of the biosensing performance was studied by successive detection of 10 μM glucose six times. A good reproducibility is acquired with the relative standard deviation (RSD) of 3.03%. Furthermore, after 15 successive detections, the current response still retains 96% of the initial value, indicating good operational stability of the as-prepared biosensor (Figure 8A). Even stored at 4 °C in a refrigerator after four weeks, the copolymer based biosensor retains 80% of the initial current response value (Figure 8B). These results demonstrate that hierarchically porous block copolymer film offers a favorable microenvironment for the immobilization of GOx and retains its high enzymatic activity.

## 4. Conclusions

In this work, block copolymer film with a hierarchically porous structure was prepared for the immobilization of enzyme. The results proved that the hierarchically porous structure offered a bio-compatible microenvironment for the immobilization of enzyme and enhanced the direct electron transfer between the enzyme and electrode surface. Then, a glucose biosensor was constructed based on the porous matrix as the electrode material. The GOx in PS-*b*-P4VP film retained its native structure and bioactivity, and hence exhibited fast amperometric response to glucose. Compared with previously reported glucose biosensor, the as-developed biosensor exhibited more surprising performances in response time (2 s), linear range (10–4500 μM), and detection limit (0.05 μM). This work illustrates that the hierarchically porous block copolymer film is a great support matrix for immobilization of enzyme and provides a potential electrode material to construct novel biosensors with excellent performance. 

## Figures and Tables

**Table 1:** Comparison of the amperometric glucose biosensor.

Electrode Taterials	Sensitivity (µA·mM^−1^·cm^−2^)	Detection Limit (µM)	Linear Range (mM)	Response Time (s)	Reference
PtNPs@SnS_2_/nafion	10.56	2.5	0.1–1	5	[26]
GA-bacteria/GDH-bacteria/MWNTs	-	40	0.1–2	5	[27]
Cu-Co-ZIFs/PGO	2426	0.15	0.0005–3.354	3	[28]
Co_3_O_4_@G	628	0.038	0.02–8	4	[29]
MnO_2_-Ag@C	127.2	0.17	0.0005–5.7	2	[30]
GNPs/GN-CS	-	1.6	0.005–0.035	5	[31]
Cage-like PbS/Nafion	11.02	0.1	0.05–1.45	7	[32]
MWCNTs-SnS_2_	21.65	4	0.02–1.95	7	[33]
ERGO-chitosan hybrid	6.82	1.7	0.02–3.2	6	[34]
CuOnanoneedle/graphene/CNF	-	0.1	1–5.3	2	[35]
PANI microtube	35.42	0.8	0.004–0.80	3	[36]
Cu_2_O/Nafion	2038.2	0.4	0.00125–0.0375	3	[37]
Co_3_O_4_-HND	708.4	0.58	0.002–6.06	2	[38]
ERGO-MWCNT	-	4.7	0.01–6.5	5	[39]
PtPd-MWCNTs	112	0.31	0.062–14.07	5	[1]
PS-*b*-P4VP film	54.74	0.05	0.01–4.5	2	this work
